# Recent Advances in 3D Cultures

**DOI:** 10.3390/ijms25084189

**Published:** 2024-04-10

**Authors:** Vittorio Picchio, Roberto Gaetani, Isotta Chimenti

**Affiliations:** 1 Department of Angio Cardio Neurology, IRCCS Neuromed, 86077 Pozzilli, Italy; vittorio.picchio@uniroma1.it; 2 Department of Molecular Medicine, Sapienza University, 00161 Roma, Italy; roberto.gaetani@uniroma1.it; 3 Department of Medical Surgical Sciences and Biotechnologies, Sapienza University, Corso della Repubblica 79, 04100 Latina, Italy

Methods and protocols for creating complex 3D cell culture systems have been rapidly advancing in the past decade from the perspective of biomaterials [1] and structural design [2]. Creating a 3D microenvironment for specific cell types in vitro allows multifaceted exchanges between cells and cell types, as well as stimuli that are functional for effectively obtaining specific phenotypes in culture and for mirroring tissue complexity in both homeostasis and pathology [3,4]. These tools can be exploited to successfully produce tissue-like structures and cellular organoids, which can be used to model the microenvironment in a physiological way, which is relevant for many applications, including drug screening [5]. They can be also used to develop tissue engineering strategies in the clinical translation path of regenerative medicine protocols [6].

The Special Issue entitled “Recent Advances in 3D Cultures” has gathered research contributions in the wide field of 3D cultures, aiming at implementing and optimizing in vitro systems representative of the tissue microenvironment in tissue homeostasis and pathology. These contributions have explored many different fields of application, including different tissues or organs, somatic and germinal cells, normal versus tumor microenvironments, and disease modeling versus advanced therapeutic or medical applications.

Tissue engineering of cartilage tissue is a promising emerging approach for the treatment of cartilage defects [7]. However, scaffold-based or microsurgery cell delivery can be difficult in these protocols. To this end, Singh D. et al. [8] explored a novel microsphere emulsion based on methacrylate resin to create porous scaffolds with adequate mechanical cues. These scaffolds were seeded with primary chondrocytes that created a complex extracellular matrix composition and tissue disks, highlighting the possible use of these protocols for cartilage tissue engineering applications.

Aging is a multifaceted progressive process, depending on endogenous and exogenous factors [9,10]. In recent years, strategies to ease and counteract senescence or even rejuvenate cells and tissues have been proposed [11,12]. Arcuri S. et al. [13] investigated the effect of extracellular vesicles (EVs) released by young cells on the cellular hallmarks of aging in senescent cells. Since it is known that the extracellular matrix (ECM) provides biomechanical stimuli directly influencing many aspects of cell behavior [14], they examined whether scaffolds based on ECM from decellularized young swine ovaries may maintain this induced rejuvenated phenotype. They showed that EVs transiently counteract aging, for example by increasing EV content of miR-200; however, when inserted in a young ECM microenvironment, this effect can be stabilized for longer, suggesting strong synergistic crosstalk between molecular and biomechanical mediators of cell senescence.

On a related topic, Hara S. et al. [15] examined the effect of a novel gel culture system made of polysaccharide gels (xanthan and locust bean gum) on oocyte maturation [16,17] and investigated the molecular pathways involved. They reported an improvement in the efficiency of developing blastocysts, with a lower DNA methylation level up to the morula stage, suggesting improved conditions compared to the standard plate culture. Transcriptomic analysis identified estradiol and TGFB1 as the responsible upstream mediators, and their addition to the medium mirrored the beneficial effects observed with gel culture at all stages, from oocytes to morulae. These results propose novel improved methods for embryo production and culture.

One of the most challenging organs to mimic is the lung [18,19]. Maurer J. et al. [20] proposed a novel complex 3D airway model for the study of respiratory diseases. Primary human bronchial epithelial cells were cultured on a collagen matrix with donor-matched bronchial fibroblasts, and characterized for properties and functions of epithelial barrier and ciliated epithelium. The optimization of the 3D setup allowed the simulation of a functional ciliated epithelium with properties of a stable barrier, with promising potential for applications in pathological modeling as well as pharmacological research.

For years, one of the main challenges in neuroscience has been the creation of reliable and representative in vitro models [21]. D’Antoni C. et al. [22] reviewed the literature on self-assembled, guided, or bio-printed brain organoids for the study of developmental or degenerative diseases. The authors discussed several protocols suitable for increasing the reproducibility and physiological significance of complex cultures, such as cerebral organoids, bio-printed brain models, and functionalized brain organoids. These systems, obtained from human induced pluripotent stem cells, mimic the human brain from many perspectives, such as histological organization and transcriptional profiles, thus representing important tools for physiopathological studies, as well as for drug testing and toxicology experiments, as valid alternatives to animal models.

Finally, another contribution to the Special Issue addressed the oncology field. Many 3D cancer in vitro models present several limitations in reproducing the complexity of the tumor ECM and how it can affect the tumor microenvironment in general [23]. La Rocca A. et al. [24] proposed a novel 3D colorectal cancer microtissue made of normal human fibroblasts seeded onto porous biodegradable gelatin microbeads cultured in a spinner flask bioreactor. This encouraged spontaneous ECM synthesis; then, colon cancer cells were dynamically seeded upon these microtissues. This model mirrored a complex microenvironment with ECM remodeling and fibroblast activation, with promising drug testing performance and further possibilities for combination with tissue-on-chip technologies.

In conclusion, systems for 3D cell culture offer many modeling options for progressing non-animal research tools and a much closer approximation to physiological conditions compared to traditional monolayer cultures. Their translational relevance is gaining momentum, with researchers recognizing their potential to bridge the gap between preclinical studies and clinical applications. Despite their promise, several challenges persist, including the need for standardization, reproducibility, and scalability across different cell types, tissues, and applications. Nevertheless, scientists across diverse disciplines are optimistic about the prospects of 3D in vitro models, looking forward to further advancements that could revolutionize biomedical research and therapeutic development.
